# A Coastal Seawater Temperature Dataset for Biogeographical Studies: Large Biases between *In Situ* and Remotely-Sensed Data Sets around the Coast of South Africa

**DOI:** 10.1371/journal.pone.0081944

**Published:** 2013-12-03

**Authors:** Albertus J. Smit, Michael Roberts, Robert J. Anderson, Francois Dufois, Sheldon F. J. Dudley, Thomas G. Bornman, Jennifer Olbers, John J. Bolton

**Affiliations:** 1 School of Life Sciences, Westville Campus, University of KwaZulu-Natal, Westville, South Africa; 2 Oceans and Coasts, Department of Environmental Affairs, Roggebaai, South Africa; 3 Department of Ichthyology and Fisheries Science, Rhodes University, Grahamstown, South Africa; 4 Biological Sciences Department and Marine Research Institute, University of Cape Town, Rondebosch, South Africa; 5 Seaweed Unit, Fisheries Branch, Department of Agriculture, Forestry and Fisheries, Roggebaai, South Africa; 6 Department of Oceanography and Marine Research Institute, University of Cape Town, Rondebosch, South Africa; 7 KwaZulu-Natal Sharks Board, Umhlanga Rocks, South Africa; 8 South African Environmental Observation Network, Grahamstown, South Africa; 9 Coastal & Marine Research Unit, Department of Botany, Nelson Mandela Metropolitan University, Port Elizabeth, South Africa; 10 Ecological Advice, Ezemvelo KZN Wildlife, Durban, South Africa; University of Vigo, Spain

## Abstract

Gridded SST products developed particularly for offshore regions are increasingly being applied close to the coast for biogeographical applications. The purpose of this paper is to demonstrate the dangers of doing so through a comparison of reprocessed MODIS Terra and Pathfinder v5.2 SSTs, both at 4 km resolution, with instrumental *in situ* temperatures taken within 400 m from the coast. We report large biases of up to +6°C in places between satellite-derived and *in situ* climatological temperatures for 87 sites spanning the entire *ca*. 2 700 km of the South African coastline. Although biases are predominantly warm (i.e. the satellite SSTs being higher), smaller or even cold biases also appear in places, especially along the southern and western coasts of the country. We also demonstrate the presence of gradients in temperature biases along shore-normal transects — generally SSTs extracted close to the shore demonstrate a smaller bias with respect to the *in situ* temperatures. Contributing towards the magnitude of the biases are factors such as SST data source, proximity to the shore, the presence/absence of upwelling cells or coastal embayments. Despite the generally large biases, from a biogeographical perspective, species distribution retains a correlative relationship with underlying spatial patterns in SST, but in order to arrive at a causal understanding of the determinants of biogeographical patterns we suggest that in shallow, inshore marine habitats, temperature is best measured directly.

## Introduction

A main determinant of biogeographical patterns and ecosystem processes is temperature [1]-[3]. The controlling effect of seawater temperature on the survival and reproduction of benthic organisms [1]-[6], and hence also patterns in the evolution and ecology of biological assemblages at regional scales [4]-[9] is well known. Despite the key role of temperature on species distribution, in many parts of the world there is a paucity of temperature profiles for the coastal zone [1]-[3], [10], [11], and several biogeographic and ecological studies provide few or no supporting temperature data [4]-[6], [12]. In such cases authors either relate their findings to known distributions of other groups of organisms or existing biogeographic divisions [7]-[9], [13]-[18] — clearly a less than ideal approach — or use data derived from satellite imagery [1], [7], [19]. Increasingly, satellite-derived SST data are being used to predict effects of climate change on the coastal marine biota in the future [20]-[23].

The need for suitable seawater temperature records has spurred the development of gridded data products spanning the satellite era (approximately the last three decades) or longer (instrumental data reaching back to 1662 in, for example, the ICOADS dataset; http://icoads.noaa.gov [24]). Numerous such products now exist and they have found widespread applications in biogeography [19] and Earth sciences [25]. For some world regions the temperature records contained in these diverse datasets have been validated against precise and accurate temperature measurements taken *in situ*: this is particularly true for offshore regions where it has been confirmed that satellite data generally accurately reflect reality [26], [27].

It is well known that satellite-derived temperature measurements may differ from *in situ* measurements taken more than a few mm below the water surface at the same location [28]-[30]. This was demonstrated in a shallow-water study of some bays on the coast of Western Australia, where satellite measurements of temperature were, perhaps not surprisingly, 1–2°C higher than data obtained *in situ* [10]. Satellite measurements also often failed to “detect ecologically important small-scale variability or provide reliable information on temperature extremes”. Findings of the Western Australian study are based on four sites that represent 1 000 km of tropical to warm temperate coast, with a maximum temperature of about 26°C in the north down to a minimum of about 15.5°C in the south: a range of just over 10°C. That similar differences exist at other geographical localities is to be expected; yet how widespread the problem is has not been well documented.

Currently no published seawater temperature climatologies exist for South Africa’s coastal zone as a whole, and in fact, few exist globally. Gridded datasets from satellite imagery have provided an important understanding of offshore oceanographic processes in southern Africa [31]. These data have the advantages that they are usually spatially complete and temporally coherent. While such data may be satisfactory for the interpretation of regional phenomena (especially where they have been validated through corresponding *in situ* data), they suffer from several drawbacks when applied to the coastal zone [32]. In this region, which at that scale is virtually the land-sea margin, the interaction of hydrodynamic and atmospheric forces create a complex system typified by large variability at smaller spatial scales than further offshore.

First, coastal features (e.g. bays, river mouths, upwelling cells) are often smaller than the highest resolution of most sea surface temperature (SST) data products [32]. Second, pixel-based atmospheric corrections developed for oceanic applications often fail or are inappropriate at the coast, and flagging techniques used to ‘de-cloud’ satellite SST data are also known to induce strong biases at the monthly scale in regions with a strong horizontal SST gradient such as upwelling systems [33]. Third, hydrodynamic regimes such as stratified water columns may break down at the coast in very shallow waters, and seawater temperatures measured there may not directly relate to SSTs measured tens of kilometers from the coast at the ocean’s surface. Fourth, missing pixels at the land/sea edge [7], [12] or ‘land bleed’ — i.e. pixels not flagged as missing but which are influenced by land temperatures ‘mixing’ with the actual sea temperatures [11], [34] — may influence temperature data. Besides the widely used Advanced Very High Resolution Radiometer (AVHRR) derived data, many other gridded products — as blends or from a single source — are available, and although each has its benefits and drawbacks, it is likely that all are affected by similar problems when they are applied to the inshore coastal zone.

Many biologists, particularly those working with photosynthetic organisms or symbioses (e.g. coral reefs), are concerned with organisms in the shallow inshore zone, close to the coast, where it has been shown that it may be inappropriate to apply gridded temperature data that have been derived from large-scale oceanographic measurements [10], [35]. In practice this zone is narrow and around the South African coast generally extends from the intertidal to a few hundred meters from the shore, and may include shallow reefs that rise into the upper mixed layer within this coastal band of water. Of course this definition is rather imprecise as far as exact limits of depth and offshore extent are concerned, as the lower depth limit is influenced by local bathymetry, turbidity, and, with respect to temperature stratification, exposure to swell and current patterns. What is important is that the shallow coastal zone is hydrodynamically complex and includes a very significant proportion of marine biodiversity, on both sandy and rocky shores [36].

The purpose of this paper is to demonstrate the dangers inherent in using remotely sensed data for regional biogeographical applications, which are particularly problematic at the coast. Temperature measurements used by biologists should be reliable, accurate and precise, and this is not usually the case. Our aims are threefold: i) to compare two commonly used satellite SST products for the South African marine coastal zone to temperature data obtained *in situ* (climatologies only); ii) to provide the most reliable and spatially complete coastal seawater temperature climatology that is possible using existing data for the entire South African coast; and iii) to relate these findings to the biogeography along the South African coast.

The approach we have used is to compare existing ‘point source’ *in situ* temperatures with satellite-derived SSTs. In this paper we use four *in situ* data sources. One set is collected by the South African Weather Service (SAWS) using hand-held mercury thermometers at sites along the coast. A second source is electronic underwater temperature recorders (UTRs) managed by the South African Departments of Environmental Affairs (DEA) and Agriculture, Forestry and Fisheries (DAFF), and Ezemvelo KwaZulu-Natal Wildlife (EKZNW). The third set is also comprised of UTR (‘gully probe’) data and was supplied by the South African Environmental Observation Network (SAEON). The fourth is KwaZulu-Natal Sharks Board (KZNSB) measurements taken using hand-held alcohol thermometers at sites on the KwaZulu-Natal (KZN) coast. We interpolate these 87 unevenly spaced data points against a set of evenly spaced localities representing the entire length of the South African coast (*ca*. 2 700 km), and compare the interpolated *in situ* data set with two sources of gridded SST: the AVHRR Pathfinder and the Moderate Resolution Imaging Spectroradiometer (MODIS) Terra products. We deliberately discriminate between SST obtained by orbiting infrared radiometers and ‘seawater temperature’ because the former is a measure of surface temperature anywhere on Earth within the 10–20 µm of the ocean’s surface [37] (here we used temperatures sampled at 5, 10, 15 and 20 km from the land-sea margin) and the latter a measure of temperature in the water column representing the upper mixed layer of the coastal region. It is our intent for the data to undergo further validation and refinement through the addition of more point-source data, so that this inshore temperature dataset may be adopted and used by the marine scientific community for diverse applications.

## Methods and Data

### Satellite temperature data

In this study we used two satellite-derived SST products: Pathfinder version 5.2 reprocessed Advanced Very High Resolution Radiometer (AVHRR) and Moderate Resolution Imaging Spectroradiometer (MODIS) Terra data — both having a grid pixel size of 4 km. The on-going AVHRR Pathfinder SST project entails re-processing of all AVHRR data from National Oceanic and Atmospheric Administration (NOAA) satellites starting in 1981 to present using the same algorithm [38]. Daytime SST data of Pathfinder version 5.2 (4 km resolution) were downloaded from the National Oceanographic Data Centre (NODC) website (http://www.nodc.noaa.gov). A quality flag of 4, considered as the lowest quality level for acceptable data [38], was imposed resulting in the lower quality data being discarded. This product is hereafter referred to as ‘Pathfinder’ data. The MODIS aboard the NASA Terra satellite(s) has been collecting data since 2000. Level-2 MODIS data were downloaded from the Ocean Colour website (http://oceancolor.gsfc.nasa.gov) and processed at a 4 km resolution using the SeaWiFS Data Analysis System (SeaDAS – http://seadas.gsfc.nasa.gov). The processing method is described in [33] and differs from the one used to produce MODIS Level-3 products accessible from the Ocean Colour Website. Only the daytime passes were processed allowing the cloud flag (CLDICE) to be used. The SSTWARN and SSTFAIL flags, used in the Level-3 Ocean Colour Website product, were not considered. We also used several other SeaDAS flags (ATMFAIL, LAND, HILT, HISOLZEN, LOWLW, MAXAERITER, ATMWARN, NAVFAIL, FILTER) to screen data. This product, hereafter referred to as ‘Reprocessed MODIS’ or simply ‘MODIS Terra’, has been previously used to study inter-annual variability in the southern Benguela system [33], [39].

**Figure 1:**
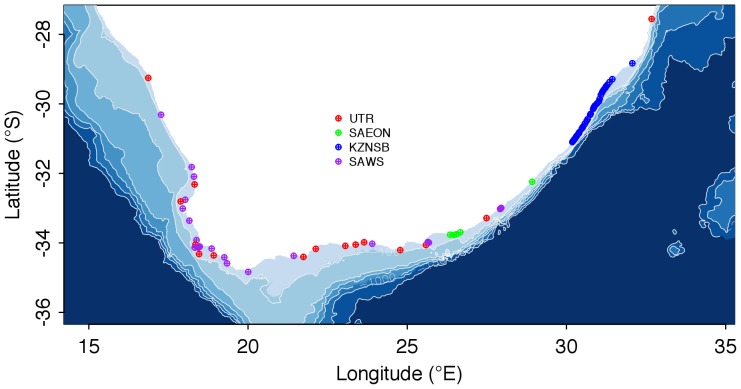
Map showing coastal sites around South Africa where seawater temperature was measured using underwater temperature recorders (UTRs) and hand-help thermometers. The legend indicates the institutions responsible for the instruments and data collections (see the Material and Methods for details). SST data were compiled in addition to the *in situ* data at each of the sites. Sites are numbered sequentially along the shore from the west coast to the east coast, and the numbers for selected sites are indicated on the figure.



The two satellite-derived SST products were extracted adjacent to the 87 *in situ* temperature measurement sites (Figure 1). With both products data were obtained along shore-normal transects at distances 5, 10, 15 and 20 km from the coast. Due to land bleed and other sources of data contamination (clouds, etc.), the extraction procedure did not rely on a single SST value. For each location the nine adjacent SST data points were selected and ‘suspicious’ values removed: we assumed that the values ‘too far’ from the mean were likely inaccurate, and therefore SST values that did not fulfill the following criteria were removed:

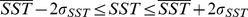



where 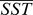 and 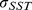 are respectively the mean and the standard deviation of the nine SST values. The remaining SST data were then averaged to obtain the final SST value for each location.

### In situ temperature data

*In situ* temperature data were obtained for 87 sites around the coast of South Africa for varying periods ranging between 1972 and 2012 (Figure 1 for sites and source of data). The size (and duration) of these datasets varied from a maximum of 13 657 (37.4 years) to a minimum of 1 131 (3.1 years) of daily temperature measurements. Temperatures were measured either manually using a hand-held thermometer or electronically using underwater temperature recorders (UTRs).

Two sources of thermometer data were used. In KwaZulu-Natal the KZNSB took temperature measurements approximately 20 cm below the sea-surface using hand-held alcohol thermometers each time shark nets were serviced (Figure 1) [40]. Service frequency was between 18–19 times per month but was less frequent during the months of June and July from the early 1990s when the nets were removed from the water for the annual sardine run [41]. The second set of thermometer data was provided by SAWS; these were measured daily at locations around the coast also using hand-held thermometers in shallow water. For many sites records date back several decades. These data are accurate to ±0.5 °C.

The DEA, DAFF and EKZN Wildlife UTRs were attached to concrete mooring blocks deployed in the shallow sub-littoral zone between depths of ∼4 and 9 m. These comprised *Starmon Mini* recorders (*Star-Oddi*, Reykjavik, Iceland) accurate to ±0.05 °C. The SAEON data were collected from shallow (1–3 m) UTRs attached to galvanised railway line segments. The UTRs used were *Onset Hobo*® U22-001 water temperature loggers that are accurate to ±0.2°C. For the temperature recorders installed in the south and west coast national parks (Storms River National Park and Table Mountain National Park) permission was obtained from the Department of Environmental Affairs; in KwaZulu-Natal the isiMangaliso Wetland Park Authority granted the necessary permits for accessing the Sodwana Bay region. The remaining 84 sites required no specific permission for obtaining temperature measurements.

All *in situ* temperature data were reduced to a time-coded, continuous series of monthly means over the duration of the sampling period for each site, and combined in one dataset of *in situ* temperature records. From this dataset a monthly temperature climatology was calculated corresponding to the satellite SST climatology. This resultant dataset was used to produce an interpolated dataset that represented temperature records at evenly spaced sites along the coast. A thin plate spline interpolation procedure was used in the ‘fields’ package under R 3.0.0 [42]. Using R 3.0.0, temperature biases were also calculated as SST_satellite_ – temperature*_in situ_* for each of the 87 measurement sites so that a warm bias indicates that the satellite-derived SSTs are higher than the corresponding *in situ* temperatures.

Both the satellite and climatological *in situ* data used in this study are available at http://www.cfoo.co.za.

## Results

### Overall South African coastal trends using in situ temperature data

**Figure 2:**
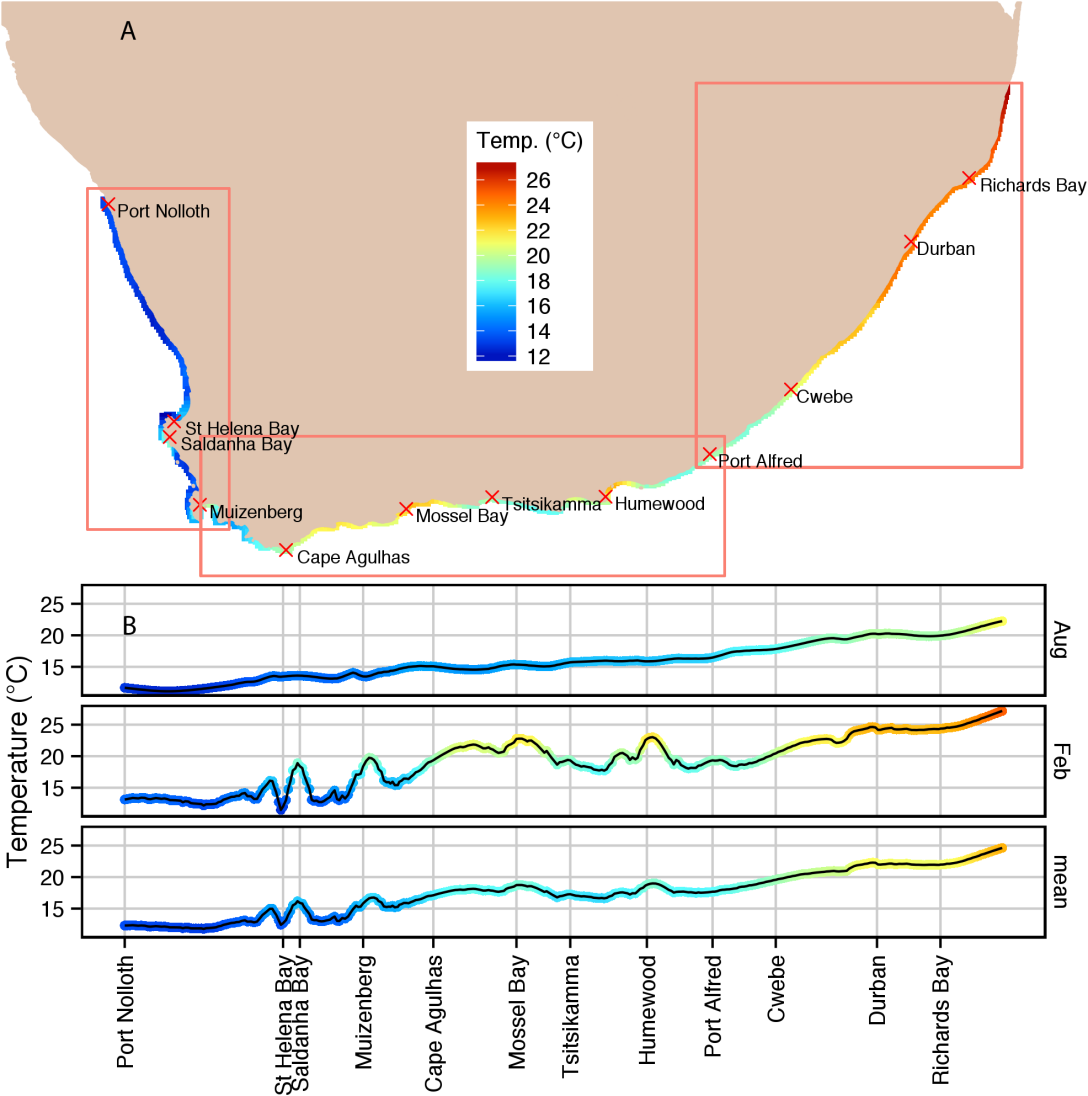
(a) Interpolated summertime inshore *in situ* temperature data for the entire coast between measurement sites 1-87 (Port Nolloth to Sodwana Bay). These same data are also plotted in the lower panel (b) to further highlight the alongshore gradients. The middle and upper panels in (b) show the seasonal mean monthly *in situ* temperature for August and February respectively representing winter and summer.

Figure 2(a), in which a colour coded shoreline is used, shows the mean monthly *in situ* temperature data for all 12 months for the entire coast between measurement sites from Port Nolloth to Sodwana Bay (sites 1 to 87). These same data are also plotted in the lower panel of Figure 2(b) to further highlight the alongshore gradients. The middle and upper panels in Figure 2(b) show the seasonal mean monthly *in situ* temperature for August (winter) and February (summer). On average (lower panel), these data indicate an increase in inshore temperatures from west to east of 13–25°C implying a difference of 12°C. In February the range increases to 13.5–27°C with a difference of 13.5°C, while in August the temperatures drop to range between 11–22.5°C (difference of 11.5°C). The August data indicate a smooth west–east temperature gradient whereas in February substantial warm fluctuations in the mean monthly inshore temperature are observed in the vicinity of St. Helena Bay (site 6; cross reference Figure 1 for site numbers) and Saldanha Bay (8) on the west coasts, and St. Francis Bay (sites 29–30) and Algoa Bay (sites 31–33) on the south coast. Other features in summer include the mean monthly temperature gradient steepening between Betty’s Bay (18) and Mossel Bay (24), and thereafter, decreasing in an eastward direction along the Tsitsikamma coast towards St. Francis Bay (sites 24–29). The *in situ* temperature gradient along the east coast also increases during the summer. Note the small decrease slightly east of Cwebe (41), and moreover that the mean monthly *in situ* temperature (lower panel) reflects all the summer gradient features with the exception of them being less dramatic.

### Regional coastal trends in in situ temperature and satellite-derived SST data

**Figure 3:**
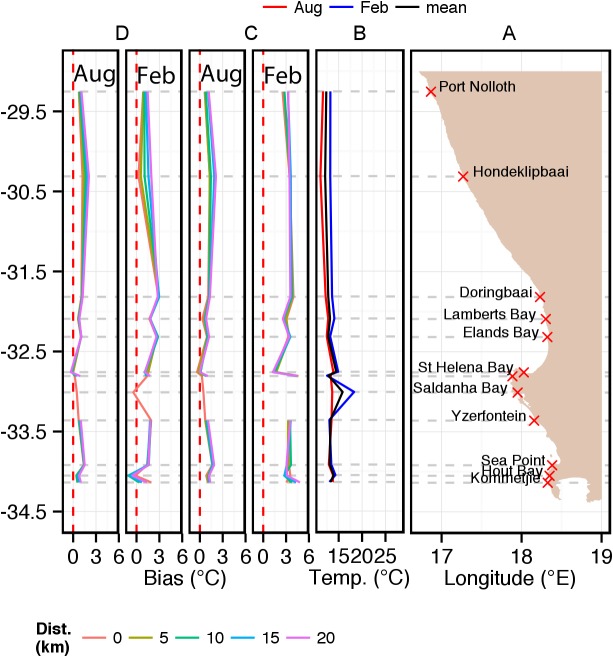
Alongshore seasonal trends (February  =  summer; August  =  winter) for *in situ* temperatures on the west coast of South Africa and concomitant biases in equivalent satellite-derived SST products: (a) indicates the locations of measurement sites; (b) *in situ* temperature with annual mean, summer and winter climatologies; (c, d) relative biases in the equivalent satellite-derived products of Pathfinder and MODIS Terra. The coloured lines in (c) and (d) depict the bias measured at 0, 5, 10, 15 and 20 km from the coast (see key).

**Figure 4:**
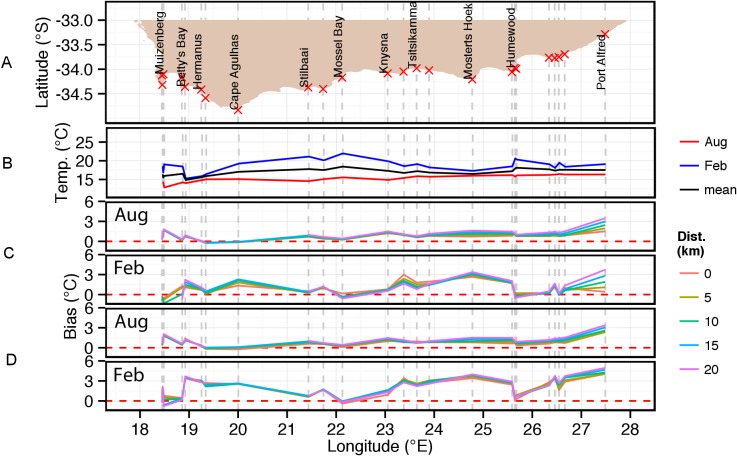
Alongshore seasonal trends (February  =  summer; August  =  winter) for *in situ* temperatures on the south coast of South Africa and concomitant biases in equivalent satellite-derived SST products: (a) indicates the locations of measurement sites; (b) *in situ* temperature with annual mean, summer and winter climatologies; (c, d) relative biases in the equivalent satellite-derived products of Pathfinder and MODIS Terra. The coloured lines in (c) and (d) depict the bias measured at 0, 5, 10, 15 and 20 km from the coast (see key).

**Figure 6:**
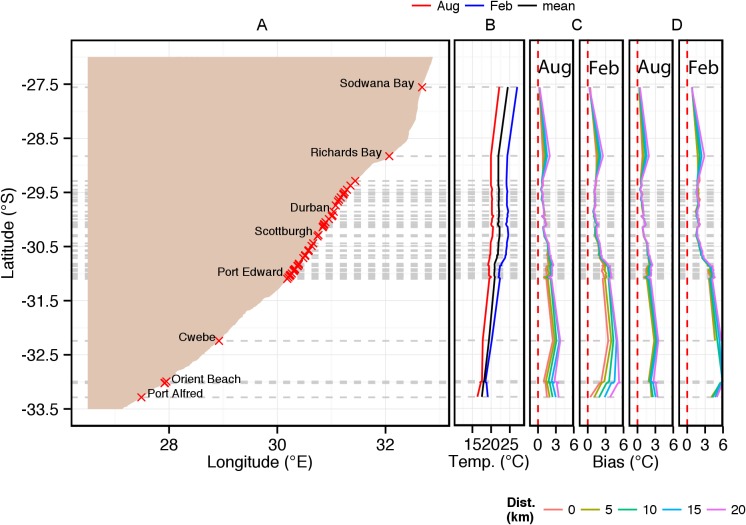
Alongshore seasonal trends (February  =  summer; August  =  winter) for *in situ* temperatures on the east coast of South Africa and concomitant biases in equivalent satellite-derived SST products: (a) indicates the locations of measurement sites; (b) *in situ* temperature with annual mean, summer and winter climatologies; (c, d) relative biases in the equivalent satellite-derived products of Pathfinder and MODIS Terra. The coloured lines in (c) and (d) depict the bias measured at 0, 5, 10, 15 and 20 km from the coast (see key).

The inshore *in situ* climatological temperature data depicted in Figure 2 are furthermore presented at greater spatial resolution in the form of west, south and east coast alongshore plots (Figures 3, 4 and 6) together with the corresponding satellite-derived SST climatological data for comparison.

**West coast (Port Nolloth to Kommetjie).** Figure 3a shows the *in situ* measurement sites along the coastline between Port Nolloth (1) and Kommetjie (12). Figure 3b depicts alongshore monthly averaged *in situ* temperatures for February, August and the mean of all 12 months. Despite the west coast being a major coastal upwelling region, it is interesting to note that the summer monthly mean temperatures along most of the coast are warmer than those of winter which instead reflect a smoother, north to south warming trend of 12–14°C. Exceptions in the summer temperature trend occur at Yzerfontein (9) and Paternoster (7) where the summer monthly mean temperature drops below that of winter. Summer monthly mean temperatures are also noticeably higher at the St. Helena Bay (6) and Saldanha Bay (8) measurement sites where temperatures are 3–5°C higher than adjacent north and south sites. These summer anomalies are possibly caused by the higher residence times for water in these embayments and consequently are subject to protracted periods of solar heating. Understandably, the mean monthly temperature (black line) therefore shows similar trends as the February *in situ* temperature data.

The Pathfinder and MODIS Terra alongshore SST data, depicted in Figures 3c and 3d respectively, show the bias relative to the *in situ* temperatures (i.e. bias temperature  =  SST_satellite_ – temperature*_in situ_*). The satellite SST data obtained along the shore-normal transects at distances 5, 10, 15 and 20 km from the coast are plotted respectively using colour lines (see key at bottom of figures). For both satellite sensors the SSTs are almost always higher (up to 5°C) than the corresponding *in situ* measurements. Exceptions however do occur — mainly in the MODIS Terra data at Saldanha Bay (8), Hout Bay (11) and Hondeklipbaai (2) in February. During August the (smaller) warm bias in the MODIS data is minimised only at St. Helena Bay (6). Given the purpose of this study, it is important to note that the Pathfinder data show the largest warm bias of two data types during summer. In winter the warm bias of the Pathfinder data is very similar to that of data obtained from MODIS Terra, also showing a minimised bias only at St. Helena Bay.

Also of interest is that both sets of satellite data indicate an offshore gradient in SST bias with the greatest bias seen at 20 km from the coast and least at the coast. This gradient is more marked in the summer MODIS Terra data north of Doringbaai (3) and south of Hout Bay (11).

**South coast (Cape Point to Port Alfred).** Similarly Figure 4a shows the *in situ* measurement sites along the coastline between Cape Point (∼13) and Port Alfred (37), and Figure 4b depicts alongshore monthly averaged *in situ* temperatures for February, August and the mean of all 12 months.

**Figure 5:**
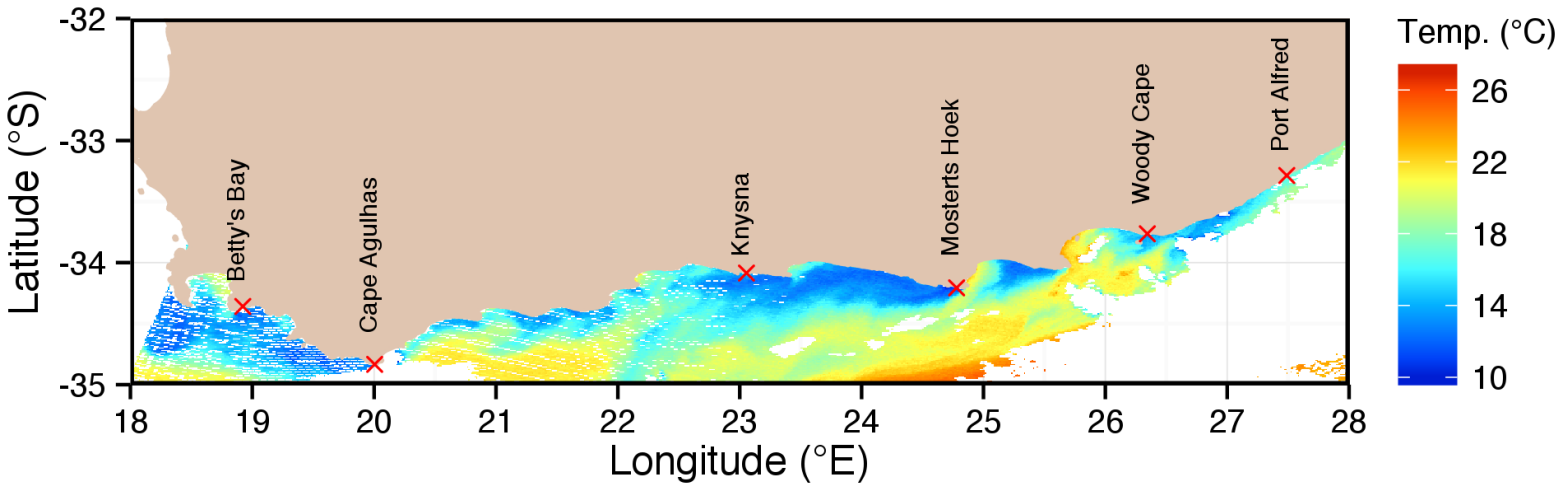
MODIS Terra satellite SST field on 4 March 2010 depicting coastal upwelling along the south coast of South Africa. Note that the strongest upwelling occurs between Betty’s Bay–Cape Agulhas, Knysna–Mosters Hoek, and Cape Padrone–Great Fish River.

Both the February and August *in situ* monthly mean temperature data in Figure 4b show a general trend of increasing temperature from west to east (∼3–4°C) although the latter is more gradual and less marked. The summer temperature gradient in contrast, due to the seawater temperatures being higher, depicts a greater fluctuation and a more complicated trend caused mainly by coastal upwelling, which is driven by the summer easterly winds. The positions of these upwelling cells are revealed in a MODIS Terra SST satellite image of the south coast on 4 March 2010 in Figure 5. Notice that the drops in the monthly mean *in situ* temperature in Figure 4b (as large as 5°C) coincide with the main coastal upwelling areas highlighted between Betty’s Bay–Cape Agulhas (18–21), Knysna–Mostert’s Hoek (25–29), and Cape Padrone–Great Fish River (34–∼36). Since the easterly winds on the south coast are a summer phenomenon, coastal upwelling along this coast is absent during winter ― in part resulting in the gradual, uncomplicated temperature trend in winter. The higher summer monthly mean temperatures correspond to those areas that do not experience the colder upwelled water, which is mostly the embayments.

As was the case with the west coast, both the MODIS Terra and Pathfinder data show the satellite-derived SSTs to be almost always warmer than the *in situ* SST measurements (Figures 4c and d). This bias is lowest during winter with the Pathfinder and MODIS Terra bias having very similar trends and magnitude, and generally <1°C. Exceptions occur in the False Bay (represented by sites 14–16) and Port Alfred (37) regions. Similar to the west coast, the warm bias is greatest during summer almost reaching 3.5°C in the Pathfinder data. In comparison the MODIS Terra data show the same trend as the Pathfinder data with very similar values where the *in situ* SST maxima occur but are ∼1°C lower in the upwelling (colder) areas. Offshore SST bias gradients are observed in both data types, both in summer and winter, with the bias gradient reversing between Ystervarkpunt (23) and Mostert’s Hoek (29) i.e. the smallest bias is inshore east of Mostert’s Hoek and west of Ystervarkpunt but largest in between. The MODIS Terra data show the largest bias gradient off Knynsa (25) and Port Alfred.

Overall, the south coast shows the greatest variability in both *in situ* temperature and satellite SST data bias.

**East coast (Orient Beach to Sodwana Bay).** As for the west and south coasts, Figure 6b shows the alongshore monthly mean *in situ* temperature for February and August, and biases relative to satellite-derived SSTs for the east coast between Orient Beach (38) near East London and Sodwana Bay (87) on the northern KZN coast. As seen in Figure 6a there is a high density of *in situ* SST measurements between Zinkwazi (85) and Port Edward (44) as a result of the shark net operations and scant data to the north and south.

Relative to the south coast the overall mean *in situ* temperatures along the east coast show smooth (quasi-linear) gradients with mean temperatures for both summer and winter decreasing steadily from north to south, i.e. 22–16°C in winter and 27–18°C in summer, implying drops of 6°C and 9°C respectively. It is noteworthy that both seasonal alongshore gradients are interrupted by stable temperatures of ∼20.5°C (August) and ∼25°C (February) in the KZN Bight and adjacent south coast between Richards Bay (86) and Park Rynie (65). This is followed to the immediate south by a short stretch of coastline displaying a steeper decreasing temperature gradient between Park Rynie and Margate (54).

As was the case with both the west coast and south coast, both satellite SST data sources on the east coast have a warm bias, which is smallest during August and largest during February. The smallest values occur at Sodwana Bay (87) in the north <0.5°C where the Agulhas Current strongly impinges on the shelf. Biases for both satellite data sources (Figures 3c and d) increase southwards to ∼1.5°C at Richards Bay (86), where shelf edge upwelling is frequent. Fairly strong offshore gradients are also found here. These biases are reduced in the KZN Bight (sites 65–86) and are coincident with the stable *in situ* temperature trend, increasing sharply towards the southern (western) portion of this coast around Port Alfred (37) and Orient Beach (38); in this region the bias is in fact stronger than anywhere else on the whole South African coast. In the case of Pathfinder (Figure 6d), biases exceed 4°C at several southern sites for which good *in situ* data are available (Margate (54) to Port Alfred (37)).

Distance offshore: South of Margate (54), distance offshore strongly affects bias in MODIS Terra data (Figure 6c) and the effect is stronger in February than in August.

**Figure 7:**
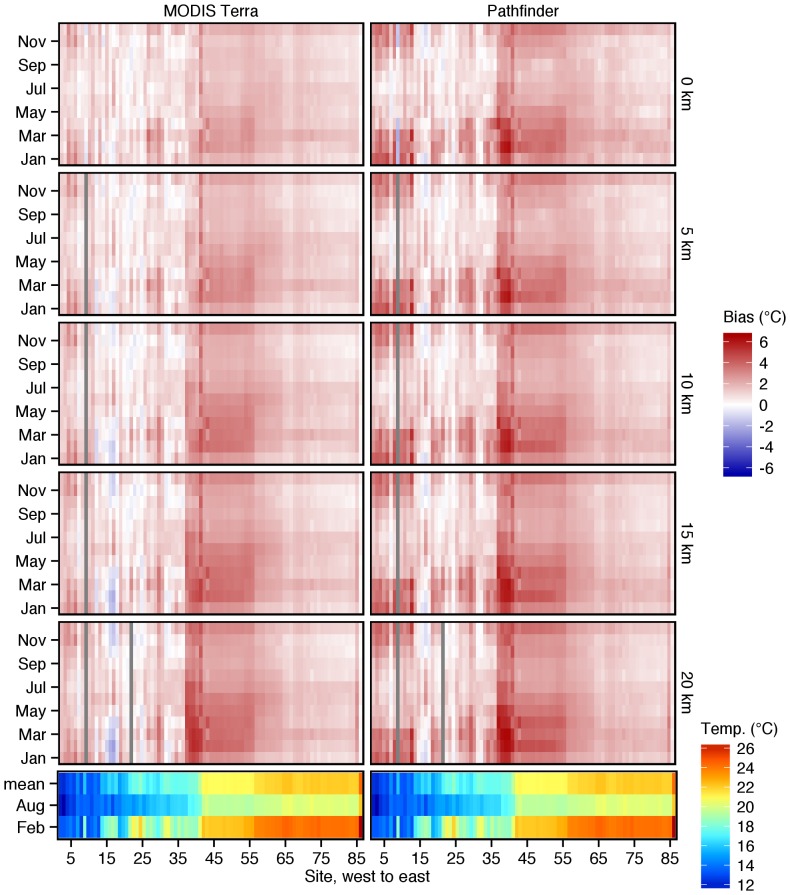
A raster image of MODIS Terra (left panels) and Pathfinder (right panels) biases with respect to *in situ* temperatures. Sites, numbered sequentially from west to east, run down the columns, and the rows within the panels depict the months of the year. The upper five panel rows show the biases at various distances from the shore (0, 5, 10, 15 and 20 km). The brightly coloured bottom row of panels is the corresponding *in situ* temperatures, with mean, February and August in the rows, and sites going down the columns. Note that the bottom left and right panels are identical, and illustrate the same temperatures that are also shown in Figures 3, 4 and 6.

**Trends in the satellite-derived warm SST bias.** Figure 7a shows the nature of the alongshore warm SST bias in both the Pathfinder and MODIS Terra data as a function of months of the year and distance offshore i.e. 0, 5, 10,15 and 20 km. See Figure 1b for conversion of number sites to place names. Scrutiny of this plot highlights several features and trends.

The first obvious feature is that, although similar ‘temporal and spatial bands’ of enhanced warm SST bias are evident in both the Pathfinder and MODIS Terra as a function of distance offshore, overall the MODIS Terra data have the lower SST bias. In the Pathfinder data the warm SST bias on the west coast between Port Nolloth and Cape Point (sites 1–14) show an increase of ∼3°C during the summer months from September to April. Maximum values of around 5°C during this time are found in the vicinity of Saldanha (8) — as also noted previously in Figure 3. The bias during the winter months (May to August) is reduced typically to <1°C. This pattern remains unchanged for all the cross-shore sites. In comparison, although noticeable throughout the year (i.e. a warm bias ∼ 1°C), the warm SST bias in the MODIS Terra data is much smaller and lacks the strong seasonal signal.

In the Pathfinder data, moving eastward, there is an abrupt change in the alongshore warm bias pattern at site Cape Point (represented by Bordjies, site 13). In fact between here and site Algoa Bay (represented by Pollock Beach and Humewood, sites 31 and 32) the bias tends to be lowest on the South African coast from Hermanus (19) to Cape St. Francis (represented by Mostert's Hoek, site 29) ― although a strong seasonal increase in the warm bias occurs during the summer months (November to April). Note that this seasonal increasing warm bias does not exactly correspond with the previously described west coast seasonal increase. Rather on the south coast the maxima in the warm bias occur in February to March as opposed to January on the west coast. As noted in Figure 4, the bias is minimal between False Bay and Hermanus (sites 14–19) and Algoa Bay (sites 31 and 32), and at times tends towards a slight negative magnitude (∼1°C) during summer. The across-shelf sites exhibit the same trend. Note that these distinct patterns are less marked in the MODIS Terra data.

Certainly in the Pathfinder data, the starkest contrast in the warm bias in the alongshore trend occurs at Port Alfred (37) where it becomes 2–6°C higher than the south coast with the larger values occurring in late summer around February and March. This elevated warm bias extends eastwards to Nahoon Beach (40) where a drop in this elevated SST bias is observed. The warm bias is relatively low eastward from here along the KZN coast until St. Michaels (56) where there is another drop in the bias, this time to around 2–3°C above the *in situ* temperatures.

**Figure 8:**
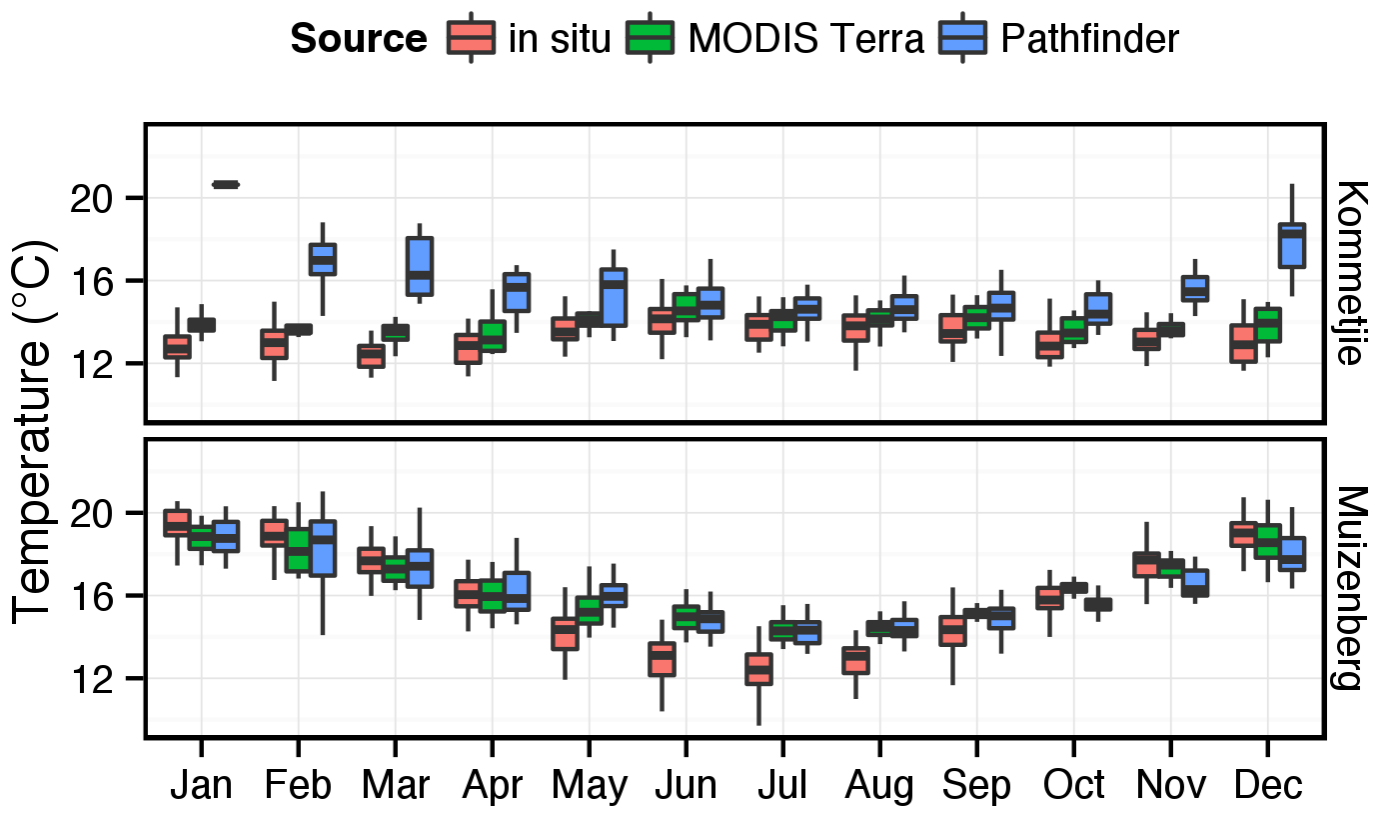
A local case study of two contrasting adjacent environments — False Bay (represented here by Muizenberg) which is an embayment mostly protected from coastal upwelling, and Kommetjie located on the exposed Atlantic side of the Cape Peninsula where wind-driven upwelling is prevalent. The *in situ* temperatures and satellite-derived SST data (MODIS Terra and Patherfinder) are presented in the form of a whisker-box plot (± 1 SD around the mean).

Figure 8 presents a local case study of two contrasting adjacent environments — Muizenberg (16), which is within an embayment mostly protected from coastal upwelling, and Kommetjie (12) located on the exposed western side of the Cape Peninsula where wind-driven upwelling is prevalent. The *in situ* and satellite-derived SST data are presented in the form of a box plot. In False Bay there is agreement between all three datasets in that they highlight the seasonal warming and cooling of the surface layer reasonably coherently. However, in winter the satellite SST data are very similar but the *in situ* temperatures are ∼2°C lower. In summer this relationship is reversed with a cold bias in the satellite SST — previously noted in Figure 7 and particularly evident between November and March when the temperature difference is ∼1°C.

By comparison the *in situ* temperature data at Kommetjie (12) show no warming of the surface layer in summer but rather a drop of 2–3°C relative to winter. Given a relatively small warm bias, the MODIS Terra SSTs follow a similar trend. The Pathfinder SSTs however starkly differ with differences ranging up to ∼6°C in midsummer.

## Discussion

The past decade has seen the consolidation of remotely-sensed SST data and blends of *in situ* and satellite data from diverse sources into a range of ‘off-the-shelf’ products for use by the marine science community. These products undergo continual revision and each iteration subjects the data to deeper scrutiny as algorithms are refined and the biases with respect to ‘sea-truthing’ datasets are understood in greater detail [26], [38], [43], [44]. Despite the many factors that can influence how well the SST products reflect the climatological reality, these SSTs are actually quite close to corresponding measurements obtained by buoys and other sources of *in situ* seawater temperatures representative of the ocean’s surface waters [26], [27], [45] — this is largely true, however, for oceanic regions.

### Regional influences on temperature biases

Although SST products developed for offshore and oceanic regions are being applied close to the coast [2], [19], [46], reports are emerging that caution the user to err away from an indiscriminate use of SST datasets in these regions. Warm and cold biases (satellite SST *minus* temperatures measured *in situ*) have been reported for several regions, e.g. Western Australia [10], [11], the west coast of the USA [1], [7], China [34], South Africa [33], and southern Belize [28]. We report here large biases — up to +6°C in places — between *in situ* and satellite-derived climatological temperatures for 87 sites spanning the entire *ca*. 2 700 km of the South African coastline, from the Benguela Current dominated west coast to the Agulhas Current influenced east coast. Biases are predominantly warm (i.e. MODIS Terra and AVHRR temperatures being warmer than corresponding *in situ* temperatures), although smaller or even cold biases also appear in places, especially along the southern and western coasts of the country.

The large spatial heterogeneity of the biases reflects largely the underlying oceanographic processes that determine the coastal seawater temperature regime. Along the east coast of South Africa (northwards of Port Alfred, site 37) the effect of the Agulhas Current is more-or-less constantly felt at the coast and upwelling is largely not present. From Port Alfred to the west upwelling is either strongly localised and intermittent (e.g. along the south coast towards Cape Agulhas, site 21) and is of a type that often occurs inshore of western boundary currents [47], or it is a seasonal feature of the nearshore, as is seen at the shoreward edge of eastern boundary currents like the Benguela Current along the west coast of South Africa [48]. This juncture between upwelling (to the west) and no upwelling (to the east), situated at Port Alfred, has a large influence on the variability and magnitude of the SST bias — it is clear that along the east coast biases are large and always positive. The seasonality of upwelling, which is strongly felt along the west coast and to a lesser extent along the south coast, is also very visible in the biases, such that cold biases are felt when upwelling is most intense during the austral summer (*ca*. September to March). The presence of embayments such as particularly False Bay (sites 16–18) also influences the bias, and in this instance results in colder SSTs relative to the *in situ* temperatures.

### Intrinsic differences between data sets

The preceding discussion cited regional influences to explain the biases between satellite and *in situ* temperatures in this study, but it is important to note that the two data sources are intrinsically different in the way in which they are obtained. Consequently the user should not be too surprised to find large discrepancies. The user should also not rely solely on the types of explanations offered above as reasons for their existence.

*In situ* data will always reflect the actual temperature of the water being measured, but instrumental differences – for example thermometer *vs*. electronic sensor – will of course have a bearing on the accuracy of the measurement. Satellite-derived measurements occur remotely and the sensor never comes into contact with the water. Here, biophysical properties (such as emitted infrared (IR) radiation in the case of MODIS Terra and the AVHRR instruments) are related through complex and sometimes inadequate algorithms to temperature [49]. We offer a few considerations that no doubt partly explain the temperature biases shown in this study.

**Skin vs. bulk measurement discrepancies.** The MODIS Terra and Pathfinder 5.2 SSTs relate to the “skin” temperature, a layer of several µm thick from which the IR photons are emitted. Both are referenced to infrared radiometric measurements made by the ship-based Marine-Atmospheric Emitted Radiance Interferometer (M-AERI) and by buoy measurements (“bulk”) at a depth of about a meter [43]. Matchups show a good agreement with temperatures derived through the M-AERI, such that the mean error, represented as MODIS *minus* M-AERI SST, is 0.20 ± 0.26°C (mean ± SD); similarly reference to buoy data is -0.07 ± 0.43°C [49]. The nominal offset between the skin and bulk SST is around 0.17°C with the skin temperature being colder, but this depends on the magnitude and direction of heat flux, and is as such highly dependent on surface winds [26]. Due to diurnal warming the MODIS daytime observations are ∼0.2°C warmer than the nighttime measurements at latitudes equivalent to those of our study region, in areas of winds greater than 3.5 m.s^−1^. At wind speeds >6 m.s^−1^ the diurnal warming signature is minimised [26].

Our instrumental seawater temperatures should represent that of the bulk seawater. The thermometer readings provided by the SAWS and KZNSB are of the top ∼20 cm, while most of the UTRs are positioned at ∼3 m (except for those at Mossel Bay, Knysna, Plettenberg Bay and Tsitsikamma, which are between 7 – 9 m). We have very good confidence that the majority of the temperatures reflect that of the upper mixed layer (see below), but there is a concern that vertical temperature stratification would likely have influenced those at the sites deeper than 3 m. However, given the often very large temperature biases observed in our study, it is not likely that skin *vs*. bulk characteristics (<0.2°C) can explain this outcome.

**Inshore hydrodynamics.** Several physical processes operate in the surface mixed layer at the coast:

- the injection of turbulence through breaking waves in the surf zone or outside of the surf zone, which increases breakdown of the mixed layer;
- convective mixing due to cooling through evaporation, which mostly occurs when the air is cold and dry, predominantly during winter months; this results in destratification of the water column vertical structure;
- mixing through velocity shear caused mostly through wind driven currents, and which again causes vertical mixing; and,
- tidal mixing, which also minimises the vertical thermal gradient.

These processes effectively homogenise the first few meters of the water column and therefore minimise the difference between the skin temperature and the deeper bulk temperature. In the region where the majority of our measurements were taken these processes reduce vertical stratification that may characterise waters further offshore, such as the regions occupied by the buoys yielding the reference bulk temperatures eluded to earlier [43]. In hydrodynamically active coastal zones, such as along much of the South African coast, the absence of shallow stratification would likely manifest in a body of water cooler (because colder, deeper water is mixed through to the surface) than the bulk surface waters of oceanic regions, to which the satellite SSTs have been referenced. The effect of upwelling should also be considered as it can tilt up the isotherms near the shore and might produce bulk water that is cooler than bulk water offshore. It should be noted, however, that the opposite effect might also manifest at the coast, i.e. that thermal heating of quiescent coastal waters might in fact be exacerbated due to the proximity to the coast. This type of thermal heating is in fact seen in embayments that are characterised by reduced water exchange, limited wave activity and hence the deepening of the thermocline (e.g. False Bay and Saldanha Bay in this study, where biases are in fact smaller).

These processes can be expected to be highly spatially and temporally variable (depending on coastal geomorphology, bathymetry, the wind regime, etc.), and we suggest this offers part of the explanation for the large alongshore variation in temperature biases between *in situ* temperatures and SSTs.

**Across-shore thermal gradients.** The horizontal variation of the surface water temperature along shore-normal transects is another important factor to consider. The proximity of all of the *in situ* temperature measurements to the SSTs is not as in an open ocean case where the reference and satellite measurements are both taken at the same location. One measurement is made at the coast (<400 m from the shore along the KZN coast and <30 m elsewhere) and the other set extends from the shore to 20 km offshore. In terms of mixing, the *in situ* measurements are highly impacted by enhanced vertical mixing as discussed earlier, while satellite measurements are less influenced by these processes. A striking feature of our data is the decreasing gradient in SST that occurs over these 20 km shore-normal transects extending from the shoreline. A general pattern that emerges, particularly along the east coast (east of Port Alfred), is that temperatures measured by satellite-borne sensors become *colder* as one approaches the shore (in Figure 7 this is indicated by a decreasing bias). This could be caused by some of the inshore hydrodynamic processes (above). Because of this one could be tempted to suggest that for coastal applications it would be better to obtain satellite SSTs as close as possible to the shoreline. However, the use of satellite SST pixels that transgress the land/sea boundary is always bad practice and this approach should not be encouraged [34]. Also, there is considerable uncertainty around the reliability of even those intact pixels that nearly touch the shore [50] and here the use of some error-correcting scheme for identifying bad pixels is also encouraged.

It is also important to note the effect of the satellite pixel dimension. Here both products are at 4 km resolution, which in most instances provide satisfactory insight into coastal SST structure, but they do often fail to resolve very fine scale thermal gradients due to upwelling cells being smaller and localised. In these instances the biases between SST and *in situ* temperatures will be large. A 1 km pixel MODIS Terra dataset (similarly reprocessed as the 4 km version) was better able to resolve the spatial nuances of the underlying surface water thermal structure but it is not presented here; biases with respect to the *in situ* data were somewhat smaller nearer the coast.

**Differences between SST sources**. We also note substantial differences between the satellite data products used. Here we use Pathfinder v5.2 (quality flag ≥4) and a version of the MODIS Terra (daytime) product that had been reprocessed for the southern African coastal region [33], [39]. The MODIS Terra derived data show a smaller bias compared to the Pathfinder equivalent, which has also been observed in other studies in the region [33], [39]. It should also be mentioned that the standard MODIS Terra Level-3 product accessible from the Ocean Colour Website fair substantially worse that the reprocessed version used here (data not shown).

Ample evidence is provided that satellite-derived SSTs are not ideal for coastal applications such as biogeographical studies (see below) due to the presence of large biases with respect to “actual” measurements acquired through hand-held thermometers and electronic temperature recorders. Contributing towards the magnitude of the biases are factors such as data source, proximity to the shore, the presence/absence of upwelling cells or coastal embayments, and various other parameters that we have not attempted to identify in this study. In the remainder of the study we apply insights gained from a climatology derived from entirely *in situ* measurements to the biogeography along the South African shore.

### Biogeographical implications

Biogeography is the study of patterns in the geographic distribution of organisms (often at the species level). The causes of these patterns are usually gradients in the physical environment, primarily temperature [2]. Because different groups of organisms have different requirements for a range of environmental conditions (not to mention the outcomes of complex biotic interactions), their distributions may not always coincide. In the marine environment the effect of depth on vertical environmental gradients, and hence on species distribution [51], must be taken into account when comparing biogeographical studies. Nevertheless, it is possible to consider the studies of various groups of marine organisms that have been done on the South African coast, in relation to the *in situ* temperature data we present here.

Our results show that *in situ* temperature data are clearly preferred for biogeographical applications at the coast, with biases in satellite data generally around 1–2°C warmer, but as high as 6°C in places in summer. An understanding of the controlling effects of temperature on the distribution of organisms can only be improved by accurate temperature measurements.

At a broad scale, the biogeographic divisions recognised along the coast of South Africa fit well with the *in situ* climatology presented here. Spalding et al. [52], whose scheme of world marine biogeographic areas is most comprehensive, consider that our coast falls within two realms: Temperate Southern Africa (from southern Angola to a region around Cape Vidal in northern KwaZulu-Natal) and the western-most edge of the Western Indo-Pacific Realm (which extends from the Cape Vidal area across the western Indian Ocean to Sumatra). The boundary between these lies between 28.5 and 29^o^S (northern KwaZulu-Natal), where our *in situ* data show a steady rise to tropical water temperatures (see later).

Within the Temperate Southern Africa realm, Spalding et al. [52] recognise three inshore marine provinces. The regions described by Spalding et al. [52] are based on many previous studies and the opinions of local experts, all of which recognise the South African west coast as part of a distinct biogeographic region that is characterised by relatively cool water that extends up the African west coast as far as northern Namibia/southern Angola [53]. This is often termed the Benguela Marine Province [52] and its southern boundary is generally agreed to be around Cape Point. The part of this Province that lies on the South African coast is characterised (except in Saldanha Bay and St. Helena Bay) by an annual mean temperature of 12–14°C and a narrow range between mean summer (e.g. February) and mean winter (e.g. August) temperatures: in other words, a very narrow temperature range when only monthly or annual means are considered. However, particularly in spring and summer, these means mask rapid and wide temperature changes that occur on a scale of days, as periods of upwelling of cold water (min. 8–9°C) alternate with static or downwelling conditions (max. 17–20°C ) [54].

This region is best termed cool temperate (as used but not defined by Emanuel et al. [13]) because it is characterised by a temperature regime that is not strictly cold-temperate. Cold-temperate is defined as having water temperatures of >10°C in the summer, but <10°C in winter (e.g. [5]). Cool temperate could therefore be defined as where mean monthly water temperatures are always >10°C and always <15°C.

Although Lombard et al. [55] describe a “South Western Cape Inshore Bioregion” from Cape Point to Cape Columbine (as distinct from a “Namaqua Bioregion” north of this), there is no evidence for this based on inshore water temperature alone and their conclusion may have been affected by their use of factors such as habitat type in their scheme. There is also no biogeographic break in seaweed distributions near Cape Columbine (JJ Bolton & RJ Anderson, unpublished data).

East of Cape Point, from 18–19^o^E, marks the start of a warm temperate region – the Agulhas Marine Province as delimited by Spalding et al. [52]. They divide this into two “ecoregions”. The most westerly of these, the Agulhas Bank Ecoregion, extends from the Cape Peninsula to an area somewhere in the Eastern Cape (former Transkei): east of this is the Natal Ecoregion, which extends to northern KwaZulu-Natal, in the region of Cape Vidal (but see later).

The boundary between the Benguela and Agulhas Marine Provinces is therefore considered by many authors to lie near Cape Point, and here the inshore water temperature regime changes fairly markedly. East of Cape Point lies False Bay, where our data show mean water temperatures to rise suddenly, from about 14–16°C, and the February mean to jump to >18°C at Muizenberg, which lies well within the bay. This boundary, which was first elucidated by Stephenson [56], is supported by studies of fish [14], invertebrates [13], marine acari [15], in the generalised scheme of Lombard et al. [55], and for seaweeds [57]-[59].

However, the area between Cape Point and Cape Agulhas is particularly interesting from a biogeographic point of view because it is characterised by a mixture of species from the Benguela Marine Province and from the warm temperate south coast east of Cape Agulhas, and is dominated by an inshore kelp bed system notably similar to that west of Cape Point [60] but distinctly different from inshore ecosystems east of Cape Agulhas. From the point of view of seaweeds, this area is considered to be a distinct overlap zone between the Benguela Marine Province and an Agulhas Marine Province that begins around Cape Agulhas [57], [58].

Differences in interpretation of this biogeographic boundary are perhaps not surprising considering that the groups of organisms in the various studies may have very different biological and dispersal characteristics. Stephenson [56] in his pioneering study of this coast, was fully aware of the “overlap” nature of this area: “The limit set […] to the west coast [Cape Point] is, however, an arbitrary one, and the true position is revealed if we ignore it for the moment […] there is a rapid falling off of west coast species in the Agulhas region ….”

The absence of kelp beds east of Cape Agulhas (but see [61] for a minor and recent exception) is not surprising, because our data show that inshore temperatures rise steadily here, and the February mean exceeds 20°C which is generally regarded as the upper limit for the distribution of the two component species, *Ecklonia maxima* and *Laminaria pallida* [57], although small sporophytes of *E. maxima* can grow in culture at 22°C [58] as can gametophytes of this kelp [62]. A further factor that might restrict these large algae is the inverse relationship between water temperature and nutrient levels. Probyn & Waldron [63] showed that above about 18°C, nitrogen concentrations are generally below 100 mmol m^−2^. This would mean that east of Cape Agulhas, nutrient levels could be too low to support kelp beds for at least some months of the year.

East of Cape Agulhas, the South African south coast is characterised by warm temperate inshore waters, with annual means around 17–18°C, but rising a little in large embayments, fairly consistent August means of 15–16°C, and February means of >20°C, except between 23–25.5^o^E where upwelling lowers the temperature (see previous discussion). These temperatures fall within the accepted definition of warm temperate, which requires at least one month of the year below 20°C and one month above 15°C [5].

The Agulhas Marine Province is notable for a wide temperature range of up to 7°C difference between February (summer) and August (winter) monthly means along most of the south coast, but this range narrows sharply east of Orient Beach/East London (see previous part of Discussion). This coincides with the next biogeographic boundary area, generally considered to be rather wide, but centred near the Mbashe River, some 100 km east of East London. While there is general agreement on the approximate position of this boundary, there is some disagreement about the biogeographic interpretation (and therefore the naming) of the next region.

Spalding et al. [52] consider the region between (approximately) Cwebe and St. Lucia to form part of the Agulhas Marine Province, but differentiate it as the Natal Ecoregion (as distinct from the south coast Agulhas bank Ecoregion). Stephenson [56] called it the Sub-Tropical East Coast Province. Emanuel et al. [13], on the basis of invertebrate distributions, call it the “Sub-Tropical East Coast”, with a northern limit near Jangamo in Mozambique, but their data included species down to 200 m depth, which makes comparisons with strictly shallow-water species difficult. Sink et al. [17], in their study of intertidal communities, accord it the status of a subtropical Natal Province (with a northern boundary near Cape Vidal), but admit that there are strong affinities with the south coast. Other authors avoid the term sub-tropical [57], [58]: it was not used by Briggs [64], and nowhere is it clearly defined.

In terms of seaweed biogeography, the region between south/central Transkei (Cwebe) and Cape St. Lucia in northern KwaZulu-Natal is clearly an overlap zone between the south coast (Agulhas Province) flora and that of the tropical Indo-West pacific Marine Province [58], [65]. There is a gradual transition between warm temperate and tropical seaweeds along the KwaZulu-Natal coast, with a fairly sharp changeover of dominant elements near Cape St. Lucia. The very small number of endemic seaweeds in this overlap zone (about three species or less than 1% of the flora) is a further reason why they did not accord it the status of a biogeographic province. While Stephenson [56] described the fauna of Natal (now KwaZulu-Natal) as subtropical, he was reserved about its biogeographic status: “… the fauna of Natal […] is also characterised by an admixture of tropical species. How strong the endemic element will prove to be […] we cannot tell, and upon this depends whether Natal can be put down as a typical subtropical fauna or one primarily of a transitional nature.” Had he collected further north than Umpangazi (north of Sodwana Bay), he may have inclined to the latter opinion.

There is general agreement that there *is* a biogeographic boundary somewhere around 29^o^E (Cwebe), whatever the adjacent biogeographic regions are called. East of here, seawater temperatures continue to rise with the increasingly strong influence of the Agulhas Current, but a very important factor may be the narrow temperature range between about 32–33^o^S, which may restrict the survival and hence dispersal of tropical species westwards and temperate species eastward.

Several studies attest to a distinct and fairly sharp biogeographic boundary between warm temperate and tropical biotas in the region of Sodwana Bay/Cape St. Lucia [17], [65], following which Spalding et al. [52] consider their Temperate Southern Africa Realm to give way here to the Indo-West Pacific Realm. Here our *in situ* data show temperature to the north steadily increasing above an annual mean of about 22°C. The critical factor is that here monthly means never fall below 20°C, the lower limit for the survival of many corals [66] and the generally accepted limiting isotherm for tropical systems [56], [64]. Although most species north of Sodwana Bay have tropical affinities, there is evidence that systems from Cape Vidal to southern Mozambique have ecologically different inshore communities to the tropical Western Indian Ocean proper [67].

The position of this boundary results largely from the coastal indentation just to the south – the KwaZulu-Natal Bight. Here, the shelf widens from less than 10 km to about 50 km, and upwelling associated with shelf-edge canyons drives water into the Bight that is cooler than that of the Agulhas Current, lowering inshore temperatures by several degrees [65].

While our evidence suggests that the satellite-derived SST data show a warm bias along the whole coastline, which differs in extent in different coastal regions, the large temperature range along the South African coastline means that the SST data will show a similar pattern to at least the major biologically determined coastal regions/provinces. Temperature changes at major biogeographic breaks, e.g. Cape Point, are of such a magnitude that they can certainly be picked up by SST data, and particularly the summer (February) monthly mean SST pattern around the coastline clearly reveals the major biogeographical breaks between west, south and east coasts, despite the biases. A danger is that these patterns are correlative rather than causative. A number of studies such as on the biogeography of seaweeds [5], [6] and sea urchins [68] use comparisons of laboratory determined temperature tolerances of organisms with coastal seawater temperature regime to predict distributions and distribution change. Biases in the temperature data caused by the use of SST, as in the current datasets, would make such studies untenable.

## Concluding Statement

Finally, concerns about temperature changes in the oceans should make the accurate monitoring of seawater temperature a priority. Our results demonstrate that in shallow, inshore marine habitats, temperature is best measured directly. We recommend that coastal nations deploy systems of underwater recorders in strategic locations, and that data are made available internationally to monitor temperatures in these highly diverse and productive environments.
